# High-sensitivity C-reactive protein and low-density lipoprotein cholesterol association with incident of cardiovascular events: Isfahan cohort study

**DOI:** 10.1186/s12872-022-02663-0

**Published:** 2022-05-25

**Authors:** Amirhossein Nafari, Noushin Mohammadifard, Fahimeh Haghighatdoost, Shima Nasirian, Jamshid Najafian, Masoumeh Sadeghi, Hamidreza Roohafza, Nizal Sarrafzadegan

**Affiliations:** 1 Hypertension Research Center, Cardiovascular Research Institute, Isfahan University of Medical Sciences, Isfahan, Iran; 2 Department of Clinical Biochemistry, Faculty of Medical Sciences, Tarbiat Modares University, Tehran, Iran; 3 Isfahan Cardiovascular Research Center, Cardiovascular Research Institute, Isfahan University of Medical Sciences, Isfahan, Iran; 4 Interventional Cardiology Research Center, Cardiovascular Research Institute, Isfahan University of Medical Sciences, Isfahan, Iran; 5 Pediatric Cardiovascular Research Center, Cardiovascular Research Institute, Isfahan University of Medical Sciences, Isfahan, Iran; 6 Cardiac Rehabilitation Research Center, Cardiovascular Research Institute, Isfahan University of Medical Sciences, Isfahan, Iran; 7 Heart Failure Research Center, Cardiovascular Research Institute, Isfahan University of Medical Sciences, Isfahan, Iran; 8 School of Population and Public Health, Faculty of Medicine, University of British Columbia, Vancouver, Canada

**Keywords:** Cardiovascular disease, High-sensitivity C-reactive protein, Low-density lipoprotein cholesterol

## Abstract

**Background:**

There are many studies on high-sensitivity C-reactive protein (hs-CRP) association with cardiovascular disease (CVD); however, just a few studies investigated whether the low-density lipoprotein cholesterol (LDL-C) could participate in hs-CRP prognostic strength. This study aimed to determine the alliance of hs-CRP and LDL-C in different concentrations in occurrence cardiovascular events in the Isfahan Cohort Study (ICS).

**Methods:**

3277 participants aged 35 and above were included in the current analysis. We evaluated the association of elevated hs-CRP levels (≥ 3 mg/dL) and CVD events including myocardial infarction, ischemic heart disease, stroke, CVD, CVD mortality, and all-cause mortality in those with LDL-C ≥ or < 130 mg/dL Cox frailty models was used to determine possible interactions.

**Results:**

In both crude and fully adjusted models, there was no significant interaction between LDL-C and hs-CRP levels with the incidence of MI, stroke, CVD mortality, and all-cause death. Neither elevated LDL-C alone nor elevated CRP alone were associated with the risk of all cardiovascular events and all-cause death. However, participants with elevated concentrations of both hs-CRP and LDL-C had a greater risk of ischemic heart disease (IHD) (hazards ratio (HR) 1.44; 95% CI 1.03–2.02) and CVD (HR 1.36; 95% CI 1.01–1.83) than those with low LDL-C and hs-CRP.

**Conclusion:**

These results indicate that despite a null association between elevated levels of CRP or LDL-C alone and CVD events, concurrent rise in LDL-C and hs-CRP levels is associated with higher risk of IHD and CVD.

## Introduction

Cardiovascular diseases (CVDs) are recognized globally as the first leading cause of death [1]. CVDs claimed the lives of 17.9 million individuals worldwide in 2019, accounting for 32% of all deaths [2]. About 31% of global deaths was attributed to CVDs in 2016 [3]. Iran was among the countries with the highest age-standardized CVD incidence with over 9000 cases per 100,000 people [4].

Various metabolic abnormalities play a crucial role in the pathogenesis of CVDs. The pathogenicity of these biochemical markers might differ from one population to another due to the differences in the ethnicity, genetic, dietary habits, environmental factors, and lifestyle variables. For instance, the Iranian population have a different lipid profile from other countries. To be more precise, the upper limits of reference values for total cholesterol (TC), triglyceride (TG), and low density lipoprotein-cholesterol (LDL-C) in Tehranian individuals are higher than most Asian countries and lower than most Western countries [5]. Therefore, it is necessary to examine the relevance of various biochemical markers in the pathogenesis of CVDs in each population separately.

In addition, vascular inflammation is an essential process in the pathophysiology of CVDs [6]. C-reactive protein (CRP), a reactant in the acute phase, is a participating factor in the systemic response to low-grade inflammation. As a direct participant in atherogenesis, CRP binds to ligands exposed to dead and damaged cells to release chemotactic factors, thereby increasing inflammatory cells' penetration [7]. Elevated high-sensitivity CRP (hs-CRP) has a prognostic association with the long‐term cardiovascular risk [8, 9]. CRP can selectively bound to LDL-C through calcium-dependent reaction and is present in atherosclerotic plaques [10, 11].

Although hs-CRP is an established risk determinant for CVDs [12], it is not clear whether lower hs-CRP concentrations can reduce cardiovascular risk independent of LDL-C. This question poses an argument, chiefly with individuals whose LDL-C levels are in normal range but still manifest high level of hs-CRP. In other words, these observations raise this question that whether LDL-C levels affect the interaction between hs-CRP and CVDs. Hence, in order to evaluate the role of hs-CRP in relation with LDL-C as a risk factor for CVDs, this study aimed to examine the association of hs-CRP with the risk of CVD in different LDL-C levels in the framework of the Isfahan Cohort Study (ICS), an ongoing prospective cohort study, with primary endpoints of CVD in Iran as a Middle East country.

## Materials and methods

### Study design and subjects

The ICS is an ongoing longitudinal cohort study established in 2001 and followed for 13 years until 2013. This study recruited 6504 participants (3168 men and 3336 women) aged 35 years and over from Isfahan (n = 2153), Arak (n = 3323) and Najafabad (n = 1028), three counties of central Iran, from 2nd January till 28th September of 2001. Sampling was done by stratified cluster random method in eligible individual including Iranian, mentally competent and not pregnant, not having inflammatory states like cancers, autoimmune disease, liver and kidney diseases. To apply stratified cluster random sampling, we stratified the subjects by their living district and selected participants from chosen clusters in each district. Interviews, laboratory measurements, and physical examinations were conducted at baseline and repeated in those without any CVD events every six years. The lifestyle factors including dietary intake, smoking and physical activity; medical history and medication use through face-to-face interviews; laboratory measurement like serum lipids, blood sugar and CRP; physical examinations were collected in 2001, 2007 and 2013. However, as hs-CRP missing data was high, we used data in 2001 and 2007 in our analysis. To identify major cardiovascular events, participants were followed up biannually. Details of study design, subjects’ recruitment and data collection methods have been described elsewhere [13].

**Fig. 1:**
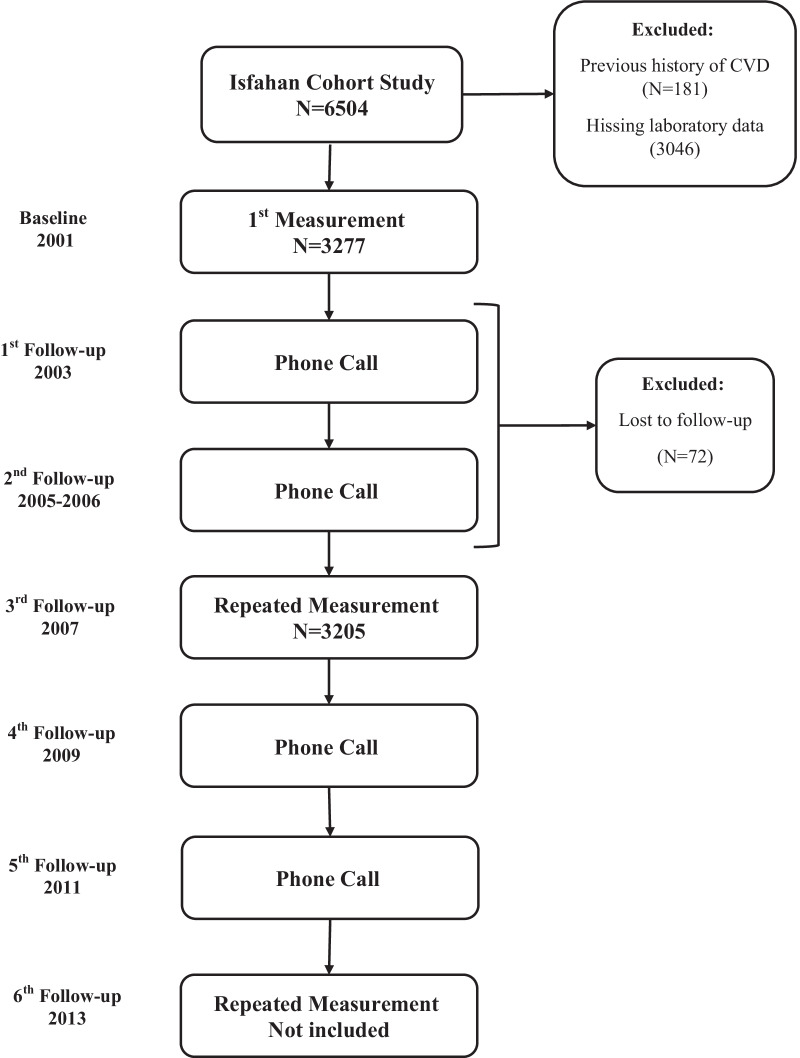
Flowchart of study design

Obtaining a written informed consent, demographic and socioeconomic status, lifestyle behaviors, all measurements and physical examination were collected at enrolment. Participants were followed every two years by telephone call and in cases of unsuccessful calls, home-interviews were done. The data also were collected at baseline survey every 6 years [14]. In the current study, we excluded subjects with a previous history of CVD (n = 181) As the hs-CRP missing data was high in 2013, we excluded this stage from the current analysis. Finally, 5432 amongst subjects in 2001 and 2007, 3277 and 3205 participants who had one and two, measurements, respectively. Therefore, we had a total of 6482 observations during 13 years of follow-up (Fig. 1). The study was approved by Ethics Committee of the Research Council of Isfahan Cardiovascular Research Center (ICRC), a WHO collaborating center in Isfahan, Iran in accordance with Declarations of Helsinki.

### Data collection

At baseline, information about demographic and socioeconomic status, lifestyle behaviors including dietary intakes, smoking status, physical activity, and medical history (e.g. the history of dyslipidemia, diabetes mellitus, hypertension, and medicine use) was collected using standard questionnaires through a 30-min home interview by trained staff [14, 15]. Anthropometric, blood pressure and laboratory measurements and physical examination were collected through participants’ attendance in the nearest health care clinic. Blood pressure was measured using calibrated mercury sphygmomanometers twice after five minutes resting, and the mean was calculated as individual’s blood pressure [16]. Height was measured using nonelastic tape without shoes to the nearest 0.5 cm, and weight was measured without shoes and with light clothing with a precision of 0.1 kg using a Seca scale. Individuals with systolic blood pressure (SBP) ≥ 140 mmHg and/or diastolic blood pressure (DBP) ≥ 90 mmHg and/or undergoing anti-hypertensive therapy were identified as hypertensive [17]. Body mass index (BMI) assessment was performed by weight division (kg) by square height (m^2^). Subjects with BMI ≥ 30 kg/m^2^ were categorized as obese [18].

### Biochemical measurements

Blood samples were gathered after 12 h fasting status and stored in laboratory of Isfahan Cardiovascular Research Institute at − 70 °C. Fasting blood glucose (FBG), serum total cholesterol (TC) and triglycerides (TG) were measured by the enzymatic method by a Hitachi auto-analyzer (Hamburg-Eppendorf Hamburg, Germany) using special kits (Immunodiagnostic, Frankfurt, Germany). High-density lipoprotein cholesterol (HDL-C) was determined enzymatically after precipitating other lipoproteins with dextran sulfate magnesium chloride [19]. The Friedewald formula was utilized to calculate LDL-C in individuals with TG < 400 mg/dL. However, direct measurement of LDL-C was performed with a turbidimetric method for those with TG ≥ 400 mg/dL [20]. In addition, apolipoprotein (apo) A and apo B were measured by photometric method [21]. Abnormal serum lipid profiles were defined based on the National Cholesterol Education Panel Adult Treatment Panel III (NCEP-ATP III) as diabetes mellitus was defined as FBG ≥ 126 mg/dL and/or taking hypoglycemic medications [22]. The participants with any of the TC ≥ 200 mg/dL, TG ≥ 150 mg/dL, LDL-C ≥ 130 mg/dL and/or HDL-C < 40 mg/dL for men and < 50 mg/dL for women were diagnosed with dyslipidemia [23]. Hs-CRP measurement was carried out by the Cobas e-411 auto analyzer immunoturbidimetric method (Roche Diagnostics International Ltd, Basel, Switzerland) [24]. Hs-CRP ≥ 3 mg/L has been indicated as a risk factor for CVD [25].

### Ascertainment of CVD events

The follow up of participants continued until the occurrence of CVD events or their last successful interview mostly before 2013, whichever happened first.

Over the 13 years of follow-up, subjects or their families had six biannual telephone interviews. Trained nurses performed structured primary interviews with three main questions; ‘is he/she alive?’, ‘has he/she been hospitalized for any reason? Specifically focus on cardiovascular and cerebrovascular events. In the occurrence of death or hospitalization, the nurses obtained the date of the events, physician diagnosis and the hospital's name during the interviews. If death occurred out of hospital, death certificates were attained from the provincial mortality database and a trained expert nurse conducted verbal autopsy including medical history, signs and symptoms before death through secondary interview with surviving family members. In the case of hospitalization events, trained nurses obtained related documents about reported hospitalization events through medical records and hospital records.

The documents related to each event were evaluated by the specialists’ outcome adjudication panel consisted of four cardiologists and two neurologists [24]. In the present study, cardiovascular events were defined as myocardial infarction (MI), fatal and non-fatal stroke (which was defined as death from cerebrovascular disease), unstable angina and sudden cardiac death (SCD), using modified criteria of WHO Expert Committee[26] Our definition for MI was based on the presence of at least two of the following criteria: 1) typical chest pain lasting more than 30 min, 2) ST (an isoelectric segment on the EKG which represents the interval between repolarization and depolarization of ventricular) elevation > 0.1 mV in at least 2 adjacent electrocardiograph leads, and 3) an increase in serum level of cardiac biomarkers (including creatine kinase (CK), creatine kinase-myoglobin binding (CK-MB), CK-MB mass (CK-MBm), or troponin (cTn). The order of diagnostic value is cTn > CK-MBm > CK-MB > CK [26]. The definition of UA required typical chest discomfort lasting more than 20 min within the 24 h preceding hospitalization and representing a change in the usual pattern of angina or pain: occurring with a crescendo pattern, being severe and described as a frank pain (REF). The diagnosis of UA might be new or be based on dynamic ST-segment or T-wave changes in at least two adjacent electrocardiogram leads [27]. Death within 1 h of onset, a witnessed cardiac arrest, or abrupt collapse not preceded by > 1 h of symptoms was considered as SCD. Moreover, the WHO stroke definition was used, that is, a rapid-onset focal neurological disorder persisting at least 24 h with probable vascular origin. We considered the first CVD events in our analysis.

### Statistical analysis

For quantitative variables, the independent sample t-test were used and manifested by mean ± standard deviation (SD). Categorical variable was analyzed using the Chi-square test and Fisher exact test and reported as numbers (percentages). We first categorized participants into four groups according to their serum levels of LDL-C (< 130 or ≥ 130 mg/dL) and hs-CRP (< 3 or ≥ 3 mg/dL). frailty cox models were fitted to examine the association between LDL-C and hs-CRP and cardiovascular events, with time to events as the time variable and the LDL-C and hs-CRP as the independent variable.The HRs and 95% CI for cardiovascular events across quartiles of LDL-C and hs-CRP were estimated based on four modeling processes: (1) crude model; (2) adjusted model by age (continuous) and also sex (male/female); (3) additionally adjusted by education (illiterate, primary school, and guidance and high school), residence area (urban/rural), smoking status (never, ex-smoker, current smoker), daily physical activity (METS minutes per week), original Global Dietary Index(score); (4) further adjustment for BMI, diabetes mellitus (yes/no) and hypertension (yes/no). To determine the linear trend of relative risks across quartiles of LDL-C and hs-CRP, we considered the LDL-C and hs-CRP as a continuous variable in Cox frailty models since we used three measurements in 2001, 2007 and 2013. Statistical analyses were carried out using Stata software, version 14 and R software version 4. In this report, p-values less than 0.05 were found statistically significant.

## Results

Overall, participants' baseline characteristics showed that from 3,277 eligible subjects for including in this study, with mean age of 50.89 ± 11.0 years, 48% had LDL-C ≥ 130 mg/dL and 53.6% had hs-CRP ≥ 3 mg/dL (Table1). The frequency of male sex was higher in individuals with low LDL-C and hs-CRP ≥ 3 (P = 0.020) compared to those with hs-CRP < 3. Also the prevalence of diabetes mellitus (P = 0.008) was higher in those with high hs-CRP compared to those with hs-CRP < 3, in high LDL-C group. However, there were no significant differences in mean age, BMI, WC and blood pressure and also the frequency of educational level, current smoking, obesity, hypertension and dyslipidemia in different categories.

**Table 1:** Baseline characteristics of participants stratified by low-density lipoprotein and high-sensitivity C—reactive protein concentrations

Characteristics	LDL-C < 130 mg/dL			LDL-C ≥ 130 mg/dL		
	hs-CRP < 3 mg/L n = 745	hs-CRP ≥ 3 mg/L n = 960	P value	hs-CRP < 3 mg/L n = 776	hs-CRP ≥ 3 mg/L n = 796	*P* value
Age (y)	49.3 ± 11.5	50 ± 11.3	0.221	52.2 ± 11.8	52.2 ± 11.8	0.982
Sex (male) (%)	51.1	56.8	0.020	44.2	44.5	0.934
Education (%)						
0–5 Years	75.8	73.2	0.428	75.6	74.9	0.921
6–12 Years	18.5	20.1		20.6	21.1	
> 12 Years	5.6	6.7		3.7	4	
Current smoker (%)	15.3	18.1	0.245	14	17	0.175
Rural (%)	31.5	22.9	< 0.0001	38.0	30.8	0.003
Obesity (BMI ≥ 30 kg/m^2^) (%)	18	18.7	0.705	22.8	24.1	0.626
Diabetes mellitus (%)	7	7.3	0.814	8.1	12.3	0.008
Hypertension (%)	22.6	22	0.873	28	28.5	0.845
Dyslipidemia (%)	75.8	75.6	0.935	98.6	99.4	0.141
Medications (%)						
Aspirin	0.0	0.0	0.319	1.0	0.1	0.035
Statins	0.1	0.2	0.717	0.1	0.4	0.329
Nicotinic acid	0.8	0.3	0.164	0.8	0.4	0.298
BMI (Kg/m^2^) mean (SD)	26.2 ± 4.5	26.1 ± 4.4	0.653	27 ± 4.46	27.1 ± 4.7	0.805
WC (cm) mean (SD)	92. 7 ± 12.6	92.5 ± 12.7	0.722	94.8 ± 13.5	94.9 ± 12.6	0.940
SBP (mmHg) mean (SD)	119.2 ± 19.6	119.4 ± 19.3	0.839	121.5 ± 21.2	122.9 ± 22.1	0.234
DBP (mmHg) mean (SD)	77.4 ± 11.4	77.8 ± 10.3	0.510	78.4 ± 12	78.7 ± 11	0.562
Physical activity	911.78 ± 549.90	933.78 ± 544.57	0.410	856.38 ± 548.79	815.23 ± 523.02	0.128
GDI	1.04 ± 0.23	1.04 ± 0.22	0.710	1.00 ± 0.25	1.01 ± 0.25	0.219

SD: standard deviation; LDL-C: low density lipoprotein cholesterol; Hs-CRP: hs-C-reactive protein; BMI: body mass index; WC: waist circumferences; SBP: systolic blood pressure; DBP: diastolic blood pressure

Table 2 presents the mean comparison of metabolic risk factors in participants with low and high hs-CRP in both LDL-C categories. The means TC (P = 0.031), LDL-C (P = 0.007) and FBG (P = 0.033) were higher in individuals having concurrent elevated levels of hs-CRP and LDL-C. While there was no significant association between other metabolic indicators in both categories.

**Table 2:** Biochemical measurement participants stratified by low-density lipoprotein and high-sensitivity c-reactive protein concentrations

Metabolic risk factors	LDL-C < 130 mg/dL			LDL-C ≥ 130 mg/dL		
	hs-CRP < 3 mg/L	hs-CRP ≥ 3 mg/L	*P* value	hs-CRP < 3 mg/L	hs-CRP ≥ 3 mg/L	*P* value
Total cholesterol (mg/dL)	179.2 ± 32.2	176.8 ± 31.3	0.143	249.4 ± 38.6	253.8 ± 41.5	0.031
Triglyceride (mg/dL)	176.2 ± 99	176.9 ± 98	0.920	193.2 ± 104	198.4 ± 102.2	0.347
LDL-C (mg/dL)	97.6 ± 23.7	95.8 ± 23.5	0.164	163.4 ± 27.2	167.3 ± 30.7	0.007
HDL-C (mg/dL)	46.4 ± 10.6	45.7 ± 10.5	0.206	47.4 ± 9.5	46.8 ± 10.1	0.250
Apolipoprotein A (mg/dL)	150.7 ± 39.2	152.8 ± 39.4	0.752	149.9 ± 32.9	157.5 ± 34.3	0.148
Apolipoprotein B (mg/dL)	107.6 ± 23.9	113.6 ± 27.9	0.839	126.1 ± 32	126.5 ± 35	0.915
FBG (mg/dL)	87.4 ± 31.6	86.3 ± 28	0.451	91.3 ± 34.7	95.4 ± 40.6	0.033

LDL-C: low density lipoprotein cholesterol; Hs-CRP: hs-C-reactive protein; HDL-C: high density lipoprotein cholesterol; FBG: fasting blood glucose

The median of follow-up was 11.25 years. During 33,121.8 person-years of follow-up, we documented 102 new cases of MI, 95 new case of stroke, 355 new cases of IHD, 450 total CVD events, 102 CVD mortality and 274 all-cause mortality. Crude and multivariable-adjusted HR (95% CIs) of CVD events for different LDL-C and hs-CRP levels are demonstrated in Table 3. There was no significant association between the interaction of LDL-C and hs-CRP levels and the incidence of MI, stroke, CVD mortality and all-cause death in both crude and fully adjusted models. Contrary, compared with reference category the risk of IHD significantly increased by 42%, 50% and 44% in individuals with both high LDL-C and hs-CRP levels in the crude and adjusted model 2 and 3 [(HR: 1.42; 95% CI 1.02–1.97) and (HR: 1.50; 95% CI 1.08–2.08) and (HR: 1.44; 95% CI 1.03–2.02, respectively). Likewise, CVD risk significantly increased among participants who had concurrent elevated levels of LDL-C and hs-CRP with the hazards of 1.43 (95% CI 1.06–1.92) in crude model, 1.43 (95% CI: 1.08–1.90) in model 2 and 1.36 (95%: 1.01, 1.83) in the fully adjusted model.

**Table 3:** Hazard ratio and confidence intervals of major cardiovascular events in high sensitivity C-reactive protein and low density lipoprotein cholesterol interaction

Events	Events rate		Crude model	Model 1^a^	Model 2^b^	Model 3^c^
	N	Person-year	HR (95% CI)	HR (95% CI)	HR (95% CI)	HR (95% CI)
Myocardial infarction						
LDL-C < 130, hs-CRP < 3	16	7644.7	1	1	1	1
LDL-C < 130, hs-CRP ≥ 3	31	9429.8	1.44 (0.80, 2.60)	1.35 (0.77, 2.36)	1.62 (0.88, 2.99)	1.67 (0.90, 3.09)
LDL-C ≥ 130, hs-CRP < 3	25	8129.7	1.19 (0.59, 2.43)	1.05 (0.54, 2.05)	1.35 (0.71, 2.55)	1.37 (0.72, 2.61)
LDL-C ≥ 130, hs-CRP ≥ 3	30	7917.6	1.41 (0.73, 2.73)	1.30 (0.70, 2.42)	1.72 (0.93, 3.20)	1.71 (0.91, 3.19)
Ischemic heart disease						
LDL-C < 130, hs-CRP < 3	66	7644.7	1	1	1	1
LDL-C < 130, hs-CRP ≥ 3	83	9429.8	1.33 (0.98, 1.82)	1.22 (0.84, 1.76)	1.14 (0.81, 1.60)	1.15 (0.81, 1.63)
LDL-C ≥ 130, hs-CRP < 3	101	8129.7	1.26 (0.88, 1.79)	1.07 (0.70, 1.63)	1.30 (0.93, 1.81)	1.29 (0.92, 1.81)
LDL-C ≥ 130, hs-CRP ≥ 3	105	7917.6	1.42 (1.02, 1.97)	1.24 (0.83, 1.84)	1.50 (1.08, 2.08)	1.44 (1.03, 2.02)
Stroke						
LDL-C < 130, hs-CRP < 3	21	7644.7	1	1	1	1
LDL-C < 130, hs-CRP ≥ 3	19	9429.8	1.21 (0.66, 2.22)	1.31 (0.60, 2.12)	0.78 (0.42, 1.45)	0.84 (0.44, 1.60)
LDL-C ≥ 130, hs-CRP < 3	26	8129.7	1.13 (0.57, 2.27)	0.95 (0.46, 1.96)	1.02 (0.57, 1.83)	1.07 (0.59, 1.96)
LDL-C ≥ 130, hs-CRP ≥ 3	29	7917.6	1.36 (0.69, 2.64)	1.21 (0.60, 2.42)	1.17 (0.66, 2.07)	1.07 (0.59, 1.93)
Cardiovascular disease						
LDL-C < 130, hs-CRP < 3	87	7644.7	1	1	1	1
LDL-C < 130, hs-CRP ≥ 3	102	9429.8	1.32 (0.99, 1.74)	1.21 (0.90, 1.62)	1.05 (0.78, 1.41)	1.07 (0.79, 1.45)
LDL-C ≥ 130, hs-CRP < 3	127	8129.7	1.26 (0.91, 1.74)	1.05 (0.76, 1.47)	1.24 (0.93, 1.65)	1.24 (0.92, 1.67)
LDL-C ≥ 130, hs-CRP ≥ 3	134	7917.6	1.43 (1.06, 1.92)	1.26 (0.92, 1.72)	1.43 (1.08, 1.90)	1.36 (1.01, 1.83)
Cardiovascular mortality						
LDL-C < 130, hs-CRP < 3	22	7644.7	1	1	1	1
LDL-C < 130, hs-CRP ≥ 3	25	9429.8	1.03 (0.55, 1.95)	0.88 (0.46, 1.69)	0.79 (0.44, 1.42)	0.87 (0.48, 1.58)
LDL-C ≥ 130, hs-CRP < 3	26	8129.7	1.21 (0.58, 2.52)	0.97 (0.46, 2.04)	0.89 (0.49, 1.57)	1.00 (0.55, 1.81)
LDL-C ≥ 130, hs-CRP ≥ 3	29	7917.6	1.14 (0.57, 2.28)	0.96 (0.47, 1.96)	1.01 (0.58, 1.76)	0.97 (0.54, 1.73)
All-cause mortality						
LDL-C < 130, hs-CRP < 3	73	2421.3	1	1	1	1
LDL-C < 130, hs-CRP ≥ 3	68	2909.3	0.73 (0.41, 1.30)	0.54 (0.36, 1.21)	0.70 (0.50, 1.47)	0.71 (0.51, 1.40)
LDL-C ≥ 130, hs-CRP < 3	60	2293.7	0.84 (0.44, 1.59)	0.62 (0.40, 1.27)	0.58 (0.41, 1.22)	0.61 (0.43, 1.36)
LDL-C ≥ 130, hs-CRP ≥ 3	73	2518.7	0.78 (0.42, 1.46)	0.59 (0.39, 1.24)	0.75 (0.54, 1.55)	0.74 (0.53, 1.44)

Model 1: Adjusted for age (yr) and sex (male/female); Model 2: Additionally, adjusted for education (illiterate and primary school/guidance and high school/university education), residence area (urban/rural), physical activity (METs minute per week), global dietary index (score) and smoking (current-smoker/ex-smoker/never smoker); Model 3: Additionally, adjusted for diabetes mellitus history (yes/no), hypertension history (yes/no), family history of cardiovascular disease (yes/no), using lipid-lowering medications c, using aspirin history (yes/no), body mass index (kg/m^2^) (2)

## Discussion

In this study, we aimed to investigate whether elevated levels of LDL-C can synergistically alter the predicting strength of hs-CRP for different CVDs. We found an increased risk of IHD and total major CVD events in participants with a higher level of both LDL-C and hs-CRP compared to those with normal levels of LDL-C and hs-CRP after adjustment for potential confounders. However, elevated levels of either LDL-C or hs-CRP were not related to the risk of any of the CVD events. In addition, there was no significant association between different high LDL-C and/or hs-CRP and MI, stroke, all-cause and CVD mortality.

The previous studies suggested that hs-CRP is a better indicator of future cardiovascular events than LDL-C [28]. Moreover, some studies demonstrated that low levels of both LDL-C and hs-CRP prevented the recurrent stroke and ischemic attack effectively [29]. CRP has been shown to accelerate atherosclerosis by directly increasing LDL-C transcytosis across endothelial cells [30].

Oxidized LDL-C (Ox-LDL-C) and L5 stimulate endothelial cells to produce CRP, which then increases endothelial cells lectin-like OX-LDL-C receptor-1 (LOX-1) expression. As a consequence, CRP imposes a positive feedback loop with LOX-1 which promotes the Ox-LDL-C or L5 uptake [31]. Ox-LDL-C, in turn, upregulates CRP expression through the IGF2 pathway in macrophages [32]. Hye Jin Yoo investigated the severity of vascular inflammation using 18F-FDG PET in healthy participants. Although the maximum target to background ratio level appeared to have the highest correlation in individuals with raised hs-CRP ≥ 2 mg/L and high LDL-C (≥ 130 mg/dL). Subjects with high hs-CRP and low LDL-C levels had a significantly higher maximum target to background ratio than those with low hs-CRP and low or high LDL-C levels [33]. It has been previously determined that the risk of CVD in Iranian populations was associated with the increasing hs-CRP [34]. Previous studies have shown the same line of results suggesting that the prognosis of CVD is more accurate by methods that examine inflammatory markers beside lipids [35].

In Penson et al.’s study, compared to subjects with low hs-CRP concentration but high concentration of LDL-C, individuals with concurrent high concentrations of hs-CRP and LDL-C showed a higher risk of CHD and stroke. However, in subjects with the same hs-CRP level and low LDL-C concentration, no significant association was observed. This study also demonstrated that the high level of hs-CRP in patients with low level of LDL-C was linked with the highest all-cause mortality risk, even more than that of in the patients with high levels of both hs-CRP and LDL-C [36].

In the You‐Cheol Hwang et al. ‘s study, cardiovascular events in patients with diabetes mellitus and recent acute coronary syndrome were correlated with elevated hs-CRP regardless of the LDL-C levels achieved by statins [37]. These findings contradict with those of ours, which might be attributable to non-lipid-lowering effects of statins on inflammation.

Our study demonstrated the same results shown in a recent study in which both LDL-C and hs-CRP continued to predict high cardiovascular risk in 4168 atherosclerosis patients [38]. In our study, the incidence of any cardiovascular events was independent of diabetes, BMI, hypertension, family history of CVD, lipid-lowering medications, and aspirin. Similarly, in Multi-Ethnic Study of Atherosclerosis with 10.3 years of follow-up, the risk of CHD was higher among participants with elevated hs-CRP and LDL-C who did not receive any lipid-lowering medication[39]. It is interesting to note that our findings revealed that adjustment for age and sex disappeared the significant association between concurrent elevated LDL-C and CRP and IHD and CVD. However, further control for demographic and lifestyle variables and also some medical conditions again lead to a higher risk for IHD and CVD in patients with both elevated LDL-C and CRP levels. On the other hand, although higher risk for IHD and CVD in patients with both elevated LDL-C and CRP levels is dependent on age and sex, demographic and lifestyle variables and medical conditions may mediate this association. This is possibly owing to the alterations in lifestyle and medical conditions by sex and age [40, 41].

In addition, Jinglin Mo et al. study showed that a lower LDL-C and hs-CRP level significantly reduced major adverse cardiac events risk [42]. Another study also demonstrated that targeting both LDL-C and hs-CRP by statin therapy in patients with acute coronary syndrome could further reduce the frequency of primary adverse cardiac events and the residual cardiovascular risk LDL-C single target [43].

### Strengths and limitations

To the best of our knowledge, this is the first longitudinal study in the Middle East that has investigated the simultaneous changes in hs-CRP and LDL-C levels in predicting cardiovascular events using repeated measurements of biochemical biomarkers over time. The current study's strengths include a vast variety of variables for which we adjusted and thoroughly conducted CVD events adjudications, as well as an appropriate sample size and a sufficient time period for CVD events incidence follow-up. Nonetheless, there were some limitations in this study. In our study, as in previous cohort studies, we could not detect causality effect. Consequently, reverse causation bias could have occurred. Our study is also susceptible to unobserved variable bias and is unable to establish causality.

## Conclusion

In conclusion, our results demonstrated that concurrent elevated levels of LDL-C and hs-CRP were associated with a greater risk of cardiovascular events, such as IHD and CVD. Therefore, it would be worthwhile taking into account both of these parameters in diagnosis, treatment and prevention approach towards cardiovascular events. In addition, further studies are required to validate the causative link of LDL-C with hs-CRP in CVDs.

## Data Availability

The datasets analyzed during the current study available from the corresponding author on reasonable request.

## Declarations

### Accordance

All methods were carried out in accordance with Declarations of Helsinki.
